# Cytokines Induced Neutrophil Extracellular Traps Formation: Implication for the Inflammatory Disease Condition

**DOI:** 10.1371/journal.pone.0048111

**Published:** 2012-10-26

**Authors:** Ravi S. Keshari, Anupam Jyoti, Megha Dubey, Nikhil Kothari, Monica Kohli, Jaishri Bogra, Manoj K. Barthwal, Madhu Dikshit

**Affiliations:** 1 Pharmacology Division, CSIR-Central Drug Research Institute, Lucknow, India; 2 Department of Anaesthesia, Chhatrapati Shahuji Maharaj Medical University, Lucknow, India; University of Bern, Switzerland

## Abstract

Neutrophils (PMNs) and cytokines have a critical role to play in host defense and systemic inflammatory response syndrome (SIRS). Neutrophil extracellular traps (NETs) have been shown to extracellularly kill pathogens, and inflammatory potential of NETs has been shown. Microbial killing inside the phagosomes or by NETs is mediated by reactive oxygen and nitrogen species (ROS/RNS). The present study was undertaken to assess circulating NETs contents and frequency of NETs generation by isolated PMNs from SIRS patients. These patients displayed significant augmentation in the circulating myeloperoxidase (MPO) activity and DNA content, while PMA stimulated PMNs from these patients, generated more free radicals and NETs. Plasma obtained from SIRS patients, if added to the PMNs isolated from healthy subjects, enhanced NETs release and free radical formation. Expressions of inflammatory cytokines (IL-1β, TNFα and IL-8) in the PMNs as well as their circulating levels were significantly augmented in SIRS subjects. Treatment of neutrophils from healthy subjects with TNFα, IL-1β, or IL-8 enhanced free radicals generation and NETs formation, which was mediated through the activation of NADPH oxidase and MPO. Pre-incubation of plasma from SIRS with TNFα, IL-1β, or IL-8 antibodies reduced the NETs release. Role of IL-1β, TNFα and IL-8 thus seems to be involved in the enhanced release of NETs in SIRS subjects.

## Introduction

NETs are produced by neutrophils to exterminate the microorganisms, which are made up of granular proteins such as elastase, cathepsin G, myeloperoxidase embedded on the back bone of nuclear DNA and histones [1], [2]. NETs formation has been documented in pre-eclamsia [3], sepsis [4], malaria [5], systemic lupus erythematosus **(**SLE) [6], and cystic fibrosis [7] patients. Aberrant NETs formation and lack of DNases to degrade NETs in the patient’s might contribute to tissue damage and autoimmune diseases [8]. LPS-activated platelets induced NET formation that resulted in liver damage [4]. Circulating free-DNA has been reported in various human diseases [9]. NETs increase in plasma may predict multi organ failure and sepsis after multiple traumas [10].

PMNs are considered major contributors to the tissue damage during inflammatory diseases. NETs contents are abundant at the site of infection and acute inflammation [1], [11], [12]. Burn, trauma, surgery and pancreatitis induce intense inflammatory response, which is defined as systemic inflammatory response syndrome (SIRS) [13], [14]. Presence of inflammatory mediators is prevalent in both infective (sepsis, malaria) and non infective pathologies (pre-eclampsia). NETs formation is an active process, is distinct from neutrophil apoptosis and necrosis [2], and is mostly mediated by ROS/RNS production involving NADPH oxidase and myeloperoxidase [2], [15], [16], [17]. NADPH-oxidase generates superoxide radicals, leading to the formation of hydrogen peroxide, which is utilized by MPO to form hypochlorite that kills bacteria, these might also lead to lipid peroxidation and membrane damage [18]. On the other hand nitric oxide (NO) by reacting with superoxide radical generates peroxynitrite radical, which is very potent oxidant. Inflammatory mediators (LPS, IL-1β, TNFα, macrophage migration inhibitory factor and IL-6) alter microvascular homeostasis [19], [20], [21], [22], blood flow, which have been associated with MODS [23]. IL-8 targets PMNs, and stimulates PMNs adhesion, degranulation, respiratory burst, and lipid mediator synthesis [24]. TNFα increases phagocytosis, degranulation and oxidative burst activity of bovine PMNs, as well as enhanced migration through endothelium due to up-regulation of endothelial adhesion molecules [25]. These mediators regulate generation of each other, such as addition of NO augments TNFα secretion from human neutrophils [26]. While peroxynitrite mediates IL-8 gene expression and IL-8 production in IL-1β and TNFα stimulated human leukocytes [27].

Most of the studies on NETs have been performed during infective conditions except pre-eclampsia. The present study was therefore undertaken in SIRS, a non-infective inflammatory group of pathologies. It was observed that the incidence of NETs release and their content was significantly more in SIRS patients. High circulating levels of IL-8, TNFα and IL-1β prompted us to evaluate their role in NETs formation, incidentally these inflammatory mediators augmented NETs release. The present study thus demonstrate role of inflammatory mediators in NETs formation, which was dependant on the enhanced free radical formation.

## Materials and Methods

### Reagents

Hoechst 33258, Sytox green, Trizol reagents were purchased from Invitrogen (Carlsbad, CA, USA). BD OptEIA™ ELISA set were from BD Biosciences (San Diego, California, USA). Antibodies and suppliers were as follows: rabbit polyclonal anti elastase (IgG, Calbiochem, San Diego, California, USA), chicken anti rabbit Alexa fluor 488 (IgG, Molecular probes, Eugene, Oregon, USA), goat polyclonal anti human IL-1β (IgG, Santa Cruz Biotechnology, Santa Cruz, California, USA), rabbit monoclonal anti human TNF-α (D5G9, IgG, Cell Signaling Technology, Denver, MA, USA), mouse monoclonal anti human IL-8 (2A2, IgG1, BD Bioscience Pharmingen, San Diego, California, USA), normal rabbit IgG, normal goat IgG and normal mouse IgG (Santa Cruz Biotechnology, Santa Cruz, California, USA). Percoll was from Amersham Biosciences Corp. (Uppsala, Sweden). Genomic DNA purification kit, 2× PCR Master Mix, 2× Maxima SYBR Green RT-PCR Master Mix were purchased from Fermentas Life Sciences (Vilnius, Lithuania). Recombinant TNF and recombinant IL-8 was purchased from R&D system (Minneapolis, MN, USA). Primers were purchased from Eurofins Genomics India Pvt Ltd (Bangalore, India). 4-Aminobenzoic acid hydrazide (ABAH), Dextran T-500, Dichlorofluorescein diacetate (DCF-DA), Diphenyleneiodonium chloride (DPI), Gelatin from cold water fish skin, Paraformaldehyde, Poly-L-Lysine, Phorbol 12-myristate 13-acetate (PMA), Recombinant IL-1β, RPMI 1640 (Phenol red free) and Tween-20 were purchased from Sigma Aldrich (St. Louis, MO, USA).

### Patient’s Selection

Blood sample was drawn from critically ill patients admitted to the trauma centre of Chhatrapati Shahuji Maharaj Medical University, Lucknow. The study was approved by the institutional review board of CSIR-Central Drug Research Institute, Lucknow and Chhatrapati Shahuji Maharaj Medical University, Lucknow and written consent was obtained from the patients’ surrogates. Kin, carers or guardians consented on the behalf of participants whose capacity to consent was reduced and institutional committee approved this consent procedure. Diagnosis of SIRS was made by the presence of two or more of the following criteria: temperature >38°C or <36°C; heart rate (HR) >90 beats/min; respiratory rate >20 breaths/min or PaCO2<32 mm Hg; or an alteration in the white blood cell count >12,000 cells/µL. Clinical findings [Acute Physiology and Chronic Health Evaluation Scores (APACHE II) and Sequential Organ system Failure Assessment score (SOFA score)] and available laboratory tests of the patients during admission to the ICU were used to classify SIRS as described previously [28]. Inclusion criteria of our study were patients of trauma, post-operative surgical patients, and patients with respiratory illness (COPD, Asthma) etc. Patients suffering from HIV, HBV, HCV infections, cancer patients and patients older than 80 years of age were excluded from our study. 20 SIRS patients (16 male, 4 female) with a mean age of 41.1±3.7 years (range 22–70) and 20 healthy volunteer blood samples were collected. The SIRS patients characteristic include mean serum creatinine levels were 3.60±0.44 mg/dl, mean SOFA score was 10.46±1.2 and mean APACHE II score was 23.38±2.1.

### Quantification of DNA and PCR of Mitochondrial and Nuclear Genes

Plasma was isolated from blood by centrifugation at 3000×g for 10 min. DNA from the 200 µl plasma sample was isolated using genomic DNA purification kit and quantified spectrophotometrically by taking absorbance at 260 and 280 nm. The constituent of plasma (extracellular) DNA was determined by amplifying two nuclear (*gapdh, actin*) and two mitochondrial (*atp6, co1*) genes. The following primers were used: **ATP synthase subunit 6 (atp6)** (5′-ATACACAACACTAAAGGACGAACCT-3′ and 5′-GAGGCTTACTAGAAGTGTGAAAACG-3′), **cytochrome oxidase c subunit 1 (co1)** (5′-GGAGTCCTAGGCACAGCTCTAA-3′ and 5′-GGAGGGTAGACTGTTCAACCTG-3′), **gapdh** (5′-CCCCTTCATTGACCTCAACTAC-3′ and 5′-GAGTCCTTCCACGATACCAAAG-3′) and **β-actin** (5-′AACTGGAACGGTGAAGGTG3′, and 5-′CTGTGTGGACTTGGGAGAGG3′). The cycling parameters were used: 95°C for 1.5 min, 55°C for 2 min, 72°C for 3 min, 30 cycles. The PCR products were separated on 3% agarose gels and visualized by ethidium bromide staining [17], [29].

### Isolation of Human Neutrophils

Peripheral blood from adult volunteers was collected by venipuncture using trisodium citrate (3.8%, 9∶1) as anticoagulant. The study was approved by the institutional review board of CSIR-Central Drug Research Institute, Lucknow and Chhatrapati Shahuji Maharaj Medical University, Lucknow; written consent was obtained from the healthy volunteers and was conducted according to the Declaration of Helsinki. Patient blood samples were collected from central venous line taking complete aseptic precaution. Blood was layered on Histopaque 1119 and centrifuged for 20 min at 800×g. The lower interphase having granulocytes was washed with RPMI 1640 medium and was loaded on the discontinuous Percoll gradients as described earlier [15], [17], [30]. Isolated PMNs were suspended in RPMI 1640 medium containing 0.5% FBS. The purity and viability of the isolated PMNs was ascertained by CD15-FITC and PI staining using FACS Caliber (Becton Dickinson, USA), which was never less than 95%.

### Measurement of MPO Enzyme Activity

MPO activity was measured in plasma and PMNs of healthy and patient samples. The PMNs were treated with hexadecyltrimethylammonium bromide containing phosphate buffer, sonicated, incubated at 37°C for 30 minutes and was centrifuged at 7000×g for 5 minutes at 4°C. 20 µl samples were mixed with phosphate buffer (pH 6.0), o-dianisidine (7.09 mM) and hydrogen peroxide (4.4 mM). Optical density was recorded at 462 nm and was converted to units of concentration by using molar extinction coefficient for oxidized o-dianisidine ε = 10,062 [M×cm]^−1^. MPO activity has been expressed as nmole/mg-protein/10 min for plasma or µmole/10^6^ cells/10 min for PMNs [28], [31].

### NETs Formation

Neutrophils (1×10^6^) were seeded on cover-slips (pre-coated with 0.001% poly L-lysine), pre-treated with NADPH oxidase inhibitor (DPI, 10 µM), or myeloperoxidase inhibitor (ABAH, 100 µM) for 15–30 min at 37°C, and then treated with PMA (10 nM), IL-8 (100 ng/ml), TNF (8 ng/ml), IL-1β (50 ng/ml) or vehicle for 120 min in a CO_2_ incubator (RS Biotech, UK) at 37°C. PMNs from healthy volunteers were incubated with plasma (20%) of healthy and SIRS subjects for 180 min. SIRS plasma were pre-treated with 1.5 µg/ml TNF-α antibody, 15 µg/ml IL-1β antibody, 5 µg/ml IL-8 antibody, 1.5 µg/ml normal rabbit IgG, 15 µg/ml normal goat IgG or 5 µg/ml normal mouse IgG for 30 minutes at 37°C [32], [33] and then incubated with neutrophils for 180 minutes at 37°C. After fixation and blocking, samples were stained overnight with 20 µg/ml of rabbit polyclonal elastase antibody and were visualized after incubation with the secondary antibody (1∶200, chicken anti-rabbit AF 488 antibody) by confocal microscope (Carl Zeiss LSM 510 META, Germany) and assessed for the incidence of NETs formation. DNA was stained with Hoechst 33258 (3 µg/ml). The percentage of NETs was assessed by quantifying the number of NETs forming neutrophils out of the total number of neutrophils as observed under high-power fields at ×400 magnification using a 40×objectives. A NET has been defined as a discrete area of bright fluorescence larger in size than neutrophils [12], [15], [17].

### Cytokine Estimation

Levels of IL-8, TNFα and IL-1β in plasma were determined by selective ELISA kit as per the manufacturer’s protocol (OptEIA; BD Biosciences, San Diego, CA, USA).

### Cytokines Expression Analysis

Total cellular RNA from PMNs was extracted by Trizol reagent. 5 µg of total RNA was digested with RNase free DNase and reverse transcribed into cDNA using RevertAid™ H minus First Strand cDNA synthesis kit using oligo (dT) primers as per manufacturer’s instruction. cDNA were amplified with PCR (Light Cycler 480 System, Roche Diagnostics). The primers used for **TNFα** (F-5′CGAGTGACAAGCCTGTAGCC3′, R- 5′TTGAAGAGGACCTGGGAGTAG3′) [34], **IL-8** (F- 5′GCCAAGGAGTGCTAAAGA3′, R-5′CTTCTCCACAACCCTCTG3′) [35], **IL-1β** (F-5′CTCTCTCACCTCTCCTACTCAC3′, R-5′ACACTGCTACTTCTTGCCCC3′) [36], [37] and **β-actin** (F-5′AACTGGAACGGTGAAGGTG3′, R-5′CTGTGTGGACTTGGGAGAGG3′) [38] amplified a 172 bp, 199 bp, 188 bp and 208 bp products respectively. Real-time RT-PCR was performed with a Maxima SYBR Green RT-PCR Kit on Roche light cycler. Reactions were 25 µl volumes including 12.5 µl of 2× Maxima SYBR Green RT-PCR Master Mix, 1 µl of cDNA template, and 0.2 µmol/l primers that were designed to amplify a part of each gene. The three-step PCR protocol applied consisted of 35 cycles of 95°C for 15 seconds; 57°C for 30 seconds for β-actin, TNF, IL-8; 60°C for 30 seconds for IL-1β; and 72°C for 30 seconds. After PCR, a melting curve analysis consisting of 1 cycle: 95°C for 0 second, 70°C for 10 second, 95°C for 0second, Cooling 1 cycle: 40°C for 3 minute was performed to demonstrate the specificity of the PCR product as a single peak. β-actin was used as reference gene for normalization.

### Free Radical Generation

PMNs (2×10^6^cells) were incubated with vehicle or various interventions [NADPH oxidase inhibitor (DPI, 10 µM), or myeloperoxidase inhibitor (ABAH, 100 µM)] for 15–30 min at 37°C, loaded with DCF-DA (10 µM) for 15 min, and then TNF (8 ng/ml), IL-1β (50 ng/ml), and IL-8 (100 ng/ml) was added. Sample was incubated for 30 min at 37°C. 10000 events were acquired to monitor free radical generation by using FACS Calibur (Becton Dickinson, USA) [15], [17], [39].

### Statistical Analysis

The normality of the distribution was tested by the Kolmogorov–Smirnov test. Results have been expressed as mean ± SEM. Comparison between two groups was performed using a Student t test and multiple comparisons were made by one-way ANOVA followed by Newman–Keuls post analysis test. Results were considered significant at p<0.05.

## Results

### Plasma DNA Content and MPO Activity

**Figure 1:**
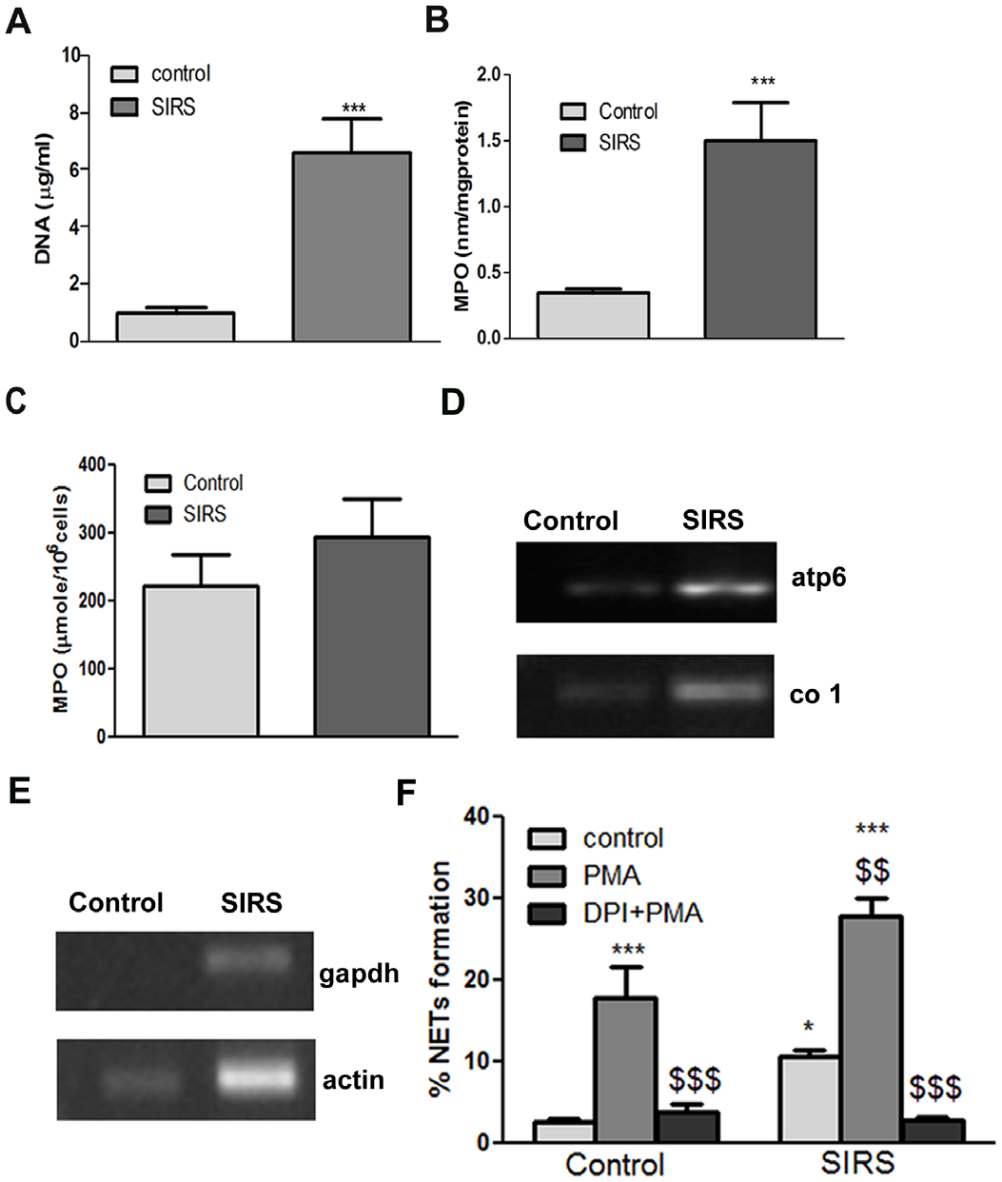
Circulating DNA content, MPO activity and NETs formation in healthy subjects and SIRS patients. (**A**) DNA content in the plasma of SIRS patients and control samples (***p<0.001 vs control). (**B**) MPO activity in plasma of SIRS patients and control subjects (***p<0.001 vs control). (**C**) MPO activity in PMNs of SIRS patients and control subject. (**D**) PCR of mitochondrial genes [ATP synthase subunit 6 (atp6), cytochrome oxidase c subunit 1 (co1)], and (**E**) nuclear genes [Glyceraldehyde-3-phosphate dehydrogenase (gapdh), and β-actin]. (**F**) Bar diagram representing NETs formation in neutrophils from healthy subjects and SIRS patients following PMA treatment (*p<0.05, ***p<0.001 vs control; ^$$^p<0.01, ^$$$^p<0.001 vs PMA stimulated cells of healthy volunteer).

DNA was isolated from the plasma of control and SIRS subjects by using genomic DNA purification kit and quantified. DNA content was significantly more in the plasma of SIRS patients (6.58±1.2 µg/ml) than control (0.94±0.30 µg/ml) **(** **Fig. 1A** **)**. MPO enzyme activity in SIRS patients plasma (1.49±0.29 nmole/mg protein/10 min) was significantly more in comparison to healthy control subjects (0.34±0.02 nmole/mg protein/10 min) **(** **Fig. 1B** **)**. MPO activity in PMNs from control (221.8±46.80 µmole/10^6^cells/10 min) and SIRS patients (295.3±54.16 µmole/10^6^cells/10 min) was however not altered significantly **(** **Fig. 1C** **)**. DNA constitutes the backbone of NETs, PCR was therefore performed to determine the source of plasma DNA by using primers for mitochondrial (*atp6* and *co1*) and nuclear (*gapdh* and *actin*) genes, which suggested presence of both nuclear and mitochondrial DNA in SIRS plasma **(** **Fig. 1D, E** **)**.

### NETs Formation in Control and SIRS Subjects

NETs were assessed as a discrete area of bright fluorescence larger in size than a normal resting neutrophil [12], which were also characterized by using immune staining of elastase and by using DNA dye [1], [2]. Spontaneous NETs release was determined in the PMNs obtained from healthy controls and SIRS patients, which was significantly more in the PMNs from SIRS patients (10±0.7%) than control subjects (2±0.3%), and it was further augmented by PMA (SIRS 28±2% vs control 18±4%). Treatment with DPI (NADPH oxidase inhibitor) completely prevented the NETs formation in both the groups, suggesting role of free radicals in NETs release from the PMNs obtained from both the subjects **(** **Fig. 1F** **)**.

Since NETs formation has been reported to be mediated through free radical generation [2], [15], [17]; free radical generation was therefore also measured in the PMNs by using DCF-DA. Free radical generation from PMNs obtained from SIRS patients was higher than the control subjects **(Fig. S1)**. NADPH oxidase (DPI) and myeloperoxidase (ABAH) inhibitors reduced PMA induced free radical generation **(Fig. S1)**, suggesting the role of these enzymes in the enhanced free radical generation.

### Effect of SIRS Plasma on NETs Formation and Free Radical Generation

**Figure 2:**
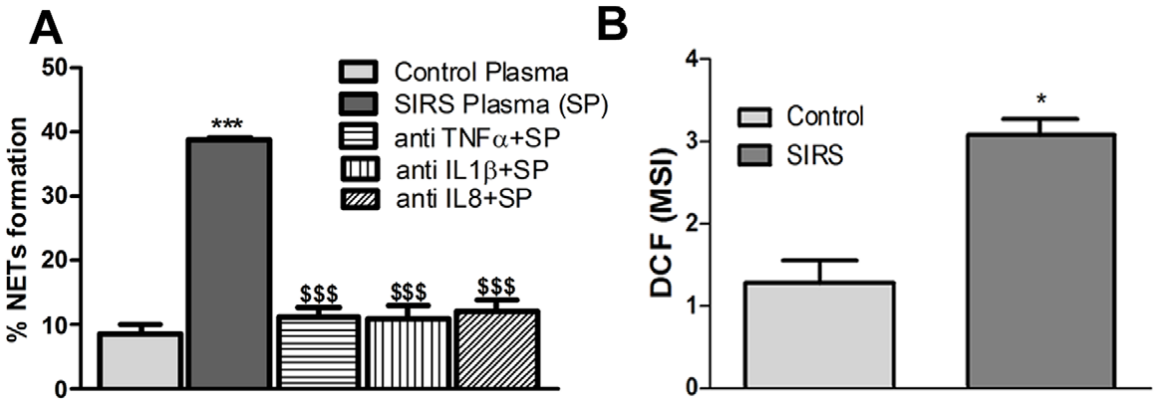
NETs formation and free radical generation. (**A**) NETs formation following incubation of PMNs from healthy subjects with plasma (20%) from healthy subjects or SIRS patients for 180 min (***p<0.001 vs control, ^$$$^p<0.001 vs SIRS). (**B**) Neutrophil free radical generation in presence of plasma (20%) from healthy subjects and SIRS patients (*p<0.05 vs control).

PMNs from healthy volunteers were incubated at 37°C for 180 min with 20% plasma from SIRS patients or healthy controls to assess their effect on NETs formation. In the presence of plasma from SIRS patients higher incidence (39±0.5%) of NETs release was observed in comparison to the presence of control plasma (8.5±1.5%). To determine if the induction of NETs formation was due to the augmented levels of cytokines, SIRS plasma (20%) was pre-incubated for 30 minute at 37°C with TNFα, IL-1β or IL-8 antibody, which led to significant reduction of NETs formation 11.4±1.5, 10.8±2.1, 12.2±1.8% respectively as compared to the non depleted plasma **(** **Fig. 2A** **)**. It was also noted that depletion/incubation with one antibody was sufficient to significantly reduce the NETs release, suggesting that the effect of cytokines might be synergistic. To further confirm, SIRS plasma (20%) was pre-incubated for 30 minute at 37°C with isotype antibody and there was no significant change in NETs formation [normal rabbit IgG (46.0±2), normal goat IgG (44±7) and normal mouse IgG (44±3)].

When PMNs from healthy volunteers were incubated with plasma (20%) from SIRS patients, free radical generation was enhanced in comparison to the control plasma **(** **Fig. 2B** **)**. PMNs incubated with depleted (pre-incubation with TNFα, IL-1β or IL-8 antibody for 30 minute at 37°C) plasma from SIRS patients for 30 mins at 37°C led to significant reduction (31%, 23% and 16% respectively) in free radical generation.

### IL-1β, TNFα and IL-8 Level in Plasma and their Expression in Neutrophils

**Figure 3:**
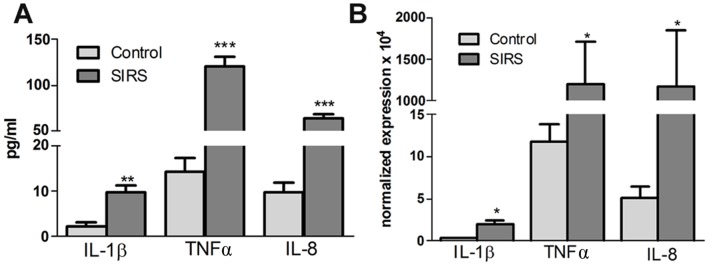
IL-8, TNFα and IL-1β levels in the plasma and their expression in PMNs. (**A**) IL-8, TNFα and IL-1β content as measured by ELISA in the plasma (**p<0.01, ***p<0.001 vs control), and (**B**) their expression in PMNs by real time RT-PCR (*p<0.05 vs control).

Circulating levels of IL-1β, TNFα and IL-8 were measured in the plasma from SIRS patients and healthy volunteers. IL-1β concentration was significantly more in the plasma from SIRS (9.71±1.4 pg/ml) than control (2.24±0.8 pg/ml) subjects. TNFα concentration was also significantly enhanced in the plasma from SIRS (120.6±11.0 pg/ml) as compared to control (14.34±2.8 pg/ml). Similarly IL-8 concentration was also augmented in the plasma from SIRS (63.06±5.5 pg/ml) as compared to control (9.7±2.0 pg/ml) **(** **Fig. 3A** **)**. Since PMNs play an important role in inflammatory pathologies, expression of these cytokines was determined by real time reverse-transcriptase PCR in the PMNs. IL-1β, TNFα and IL-8 expression was significantly enhanced in PMNs from SIRS (1.92±0.4, 1193±513.0, 1174±669.8) than control (0.35±0.03, 11.79±1.9, 5.150±1.2) subjects respectively **(** **Fig. 3B** **)**.

### IL-8, TNFα, and IL-1β Induced NETs Formation and Free Radical Generation

Since IL-8, TNFα and IL-1β concentration was significantly more in SIRS plasma than control **(** **Fig. 3A** **).** Effect of these cytokines was therefore studied on free radical generation and NETs formation. Resting neutrophils **(Fig. S3)** stained with elastase antibody (green) and Hoechst 33258 (DNA binding dye, blue) demonstrated punctated patterns of elastase distribution and multi-lobed nuclei indicating clearly the nuclear and granular components. PMNs incubated with IL-8, TNFα or IL-1β for 120 min released NETs, which were inhibited by the NADPH oxidase and myeloperoxidase inhibitor **(Fig. S2A, S3)**. PMNs incubated with IL-8, TNFα, and IL-1β induced free radical generations which were inhibited by NADPH oxidase and myeloperoxidase inhibitor suggesting that NETs formation by these interventions was mediated through free radical generated from NADPH oxidase and myeloperoxidase **(Fig. S2B)**.

## Discussion

NETs have been identified as important components of innate defense system and have been proposed to limit the spreading of microbial pathogens [1]. A significant increase in DNA and MPO content in the plasma of SIRS subjects, suggests presence of NETs contents in the plasma of SIRS subjects **(** **Fig. 1A, B** **)**. MPO activation is necessary for the formation of NETs [15], [17] **(Fig. S2A)** and circulating antibodies to MPO were associated with glomerulonephritis and systemic vasculitis [40]. Deposition of NETs initiates inflammatory response in the kidney, while in individuals with small-vessel vasculitis circulating MPO-DNA complexes triggered vasculitis and promoted autoimmune response [41]. NETs contains active MPO [42] and MPO-H_2_O_2_-Cl^-^ system generates oxidant species, which might modify macromolecules leading to the bystander tissue damage and subsequent disease progression. Presence of luminal NETs [43] and role of MPO/H_2_O_2_/Cl^–^ system in generation of modified LDL contributes to early stage of atherosclerosis [**44**]. Involvement of MPO has also been reported in various diseases like ischemia-reperfusion injury, severe sepsis, acute lung injury (ALI), and acute respiratory distress syndrome (ARDS) [45]. Moreover, impairment of NETs degradation due to circulating DNase1 inhibitors, or physical protection of NETs has been found in systemic lupus erythematosus (SLE) patients [6]. Recently cytotoxic role of NETs associated histones on endothelium and epithelium cell death in lung tissue destruction has been reported [46]. These factors thus might be responsible for the progression of disease following NETs formation.

NETs are made up of chromatin, elastase and cathepsin G, which mediate microbial killing. Pathological role of these serine proteases in inflammatory disorders has also been attributed to their ability to break down connective tissue components by generating proinflammatory peptides and by enhancing expression of proinflammatory cytokines. Neutrophil elastase is associated with microvascular injury, endothelial damage, increased capillary permeability and interstitial edema [47]. IL-8 induces elastase release in a concentration dependent manner in cytochalasin B treated PMNs and IL-1β was found to prime this release [48]. We have recently demonstrated pro-inflammatory potential of NETs [17], while this study supports role of pro-inflammatory cytokines in NETs formation. Spontaneous NETs formation in SIRS patient neutrophils was also more **(** **Fig. 1F** **)**. In the present study addition of SIRS patients plasma to the resting PMNs, led to the release of NETs, suggesting presence of NETs inducing factors in the patients plasma **(** **Fig. 2A** **)**. Role of TNFα is well documented in sepsis and has also been shown in SIRS [49]. Interestingly, addition of TNFα, IL-1β or IL-8 enhanced NETs formation and pre-treatment of SIRS patients’ plasma with their antibodies, significantly reduced NETs release and free radical generation, suggesting that neutralization of cytokines could be a useful approach for NETs mediated diseases. As IL-8 neutralizing antibody has already been found to prevent tissue damage in glomerulonephritis [50].

Many mediators such as TNFα, reactive oxygen species, NO have been speculated to cause SIRS and multiple organ injury/dysfunction syndrome [51]. TNFα, IL-1β, and IL-8, are potent activators of PMN functions. Significant increase in the circulating levels of pro-inflammatory cytokines such as TNFα, IL-8 and IL-1β as well as augmentation in their expression was observed in SIRS patients **(** **Fig. 3A & 3B** **)**. Recombinant human TNFα stimulated neutrophil respiratory burst, lysozyme release and significantly elevated the neutrophil respiratory response to fMLP [52]. Recombinant IL-1β also primes human PMNs for myeloperoxidase release [53], amplified PMNs superoxide production and PMNs spreading [54]. Cytokines also delay neutrophil apoptosis, which seems to be important for the regulation of host resistance and inflammation [55]. We and others [56] have found significant increase in free radical generation in neutrophils from SIRS patients **(Fig. S1)**. The major free radical generating enzymes in neutrophils include NADPH oxidase and myeloperoxidase, moreover enhanced DCF fluorescence [57] was reduced in the presence of their inhibitors, suggesting their role in free radical generation **(Fig. S2B**). TNFα, IL-1β, and IL-8, mediated enhanced free radical generation seems to be associated with augmented NETs formation in SIRS patients.

Recent study from this lab has demonstrated increase in nitrite in SIRS subjects and role of active nitrogen molecules in the progression of septic shock [58]. NO derived nitrate anion contributed to the endotoxic shock and multiple organ injury [59]. PMN generate nitric oxide [60], which by reacting with superoxide form the peroxynitrite. DCF binds to the hydrogen peroxide (H_2_O_2_), hydroxyl radical as well as peroxynitrite [61]. The specificity of DCF and various radical species generated in PMNs has been discussed earlier [15], [57]. ONOO**^-^** mediated IL-8 release was reported in human leukocytes challenged with LPS, TNF-α, or IL-1β [27], [62]. IL-8 has a pivotal role in the recruitment and activation of neutrophils and monocytes in various experimental models of inflammation [50]. These cytokines thus seem to play an important role in SIRS by augmenting NETs release mediated by enhanced formation of various reactive oxygen and nitrogen species. These species and NETs in turn might be augmenting cytokine production, suggesting towards a positive loop to sustain and propagate the inflammatory condition.

Results obtained in the present study demonstrate the ability of TNFα, IL-1β, and IL-8 in eliciting neutrophils oxidative burst and NETs formation. Elevated levels of these cytokines in the circulation, seems to be involved in the pathology of SIRS.

## Supporting Information

Figure S1 **Free radical generation in presence of PMA.** Bar diagram representing free radical generation as determined by DCF-DA oxidation in the presence of DPI and ABAH (***p<0.001 vs control; ^$$^p<0.01, ^$$$^p<0.001 vs PMA stimulated cells).
(TIF)

Figure S2 **IL-8, TNFα or IL-1β induced NETs release and free radical generation.** **(A)** Bar diagram representing NETs release following stimulation of PMNs from healthy subjects with recombinant IL-8, TNFα or IL-1β (***p<0.001 vs control; ^$$$^p<0.001 vs stimulator). **(B)** Bar diagram representing free radical generation following stimulation with recombinant IL-8, TNFα or IL-1β (**p<0.01, ***p<0.001 vs control; ^$^p<0.05, ^$$^p<0.01 vs stimulator).
(TIF)

Figure S3 **Immuno-histochemical characterization of IL-8, TNF or IL-1β induced NETs release in neutrophils.** Resting neutrophils stained with elastase antibody conjugated with AF 488 (green) and Hoechst 33258 (blue) showing multilobed nuclei and punctate elastase. Neutrophils treated with IL-8, TNFα or IL-1β led to the formation of NETs (Bar 10 µm).
(TIF)
