# Supplementary figures and images for: Cytokines Induced Neutrophil Extracellular Traps Formation: Implication for the Inflammatory Disease Condition
Source: PLoS One. 2012 Oct 26;7(10):e48111. doi: 10.1371/journal.pone.0048111 (PMC3482178; doi:10.1371/journal.pone.0048111)

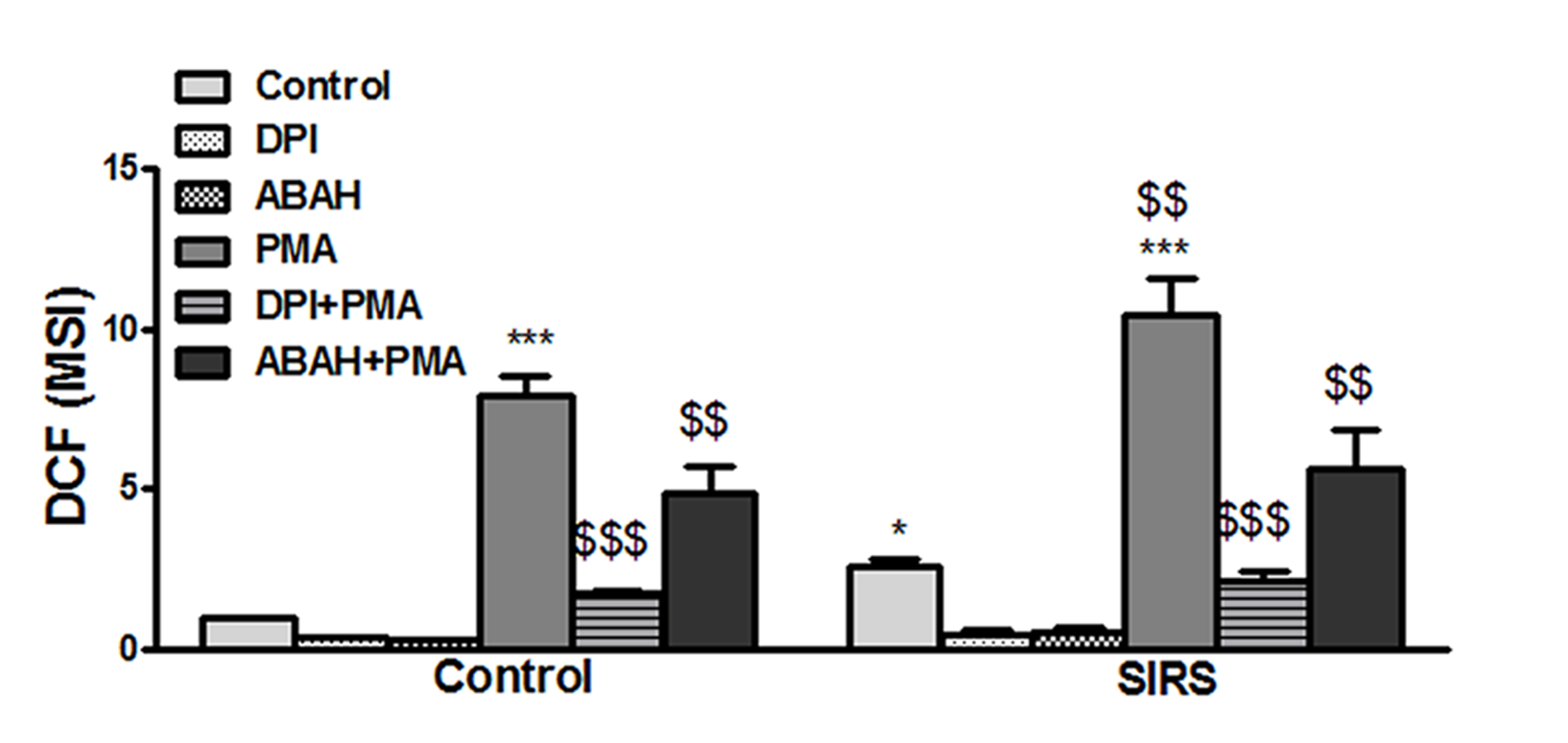

Supplement: Figure S1 — Free radical generation in presence of PMA. Bar diagram representing free radical generation as determined by DCF-DA oxidation in the presence of DPI and ABAH (***p<0.001 vs control; $$p<0.01, $$$p<0.001 vs PMA stimulated cells). (TIF) [file pone.0048111.s001.tif]

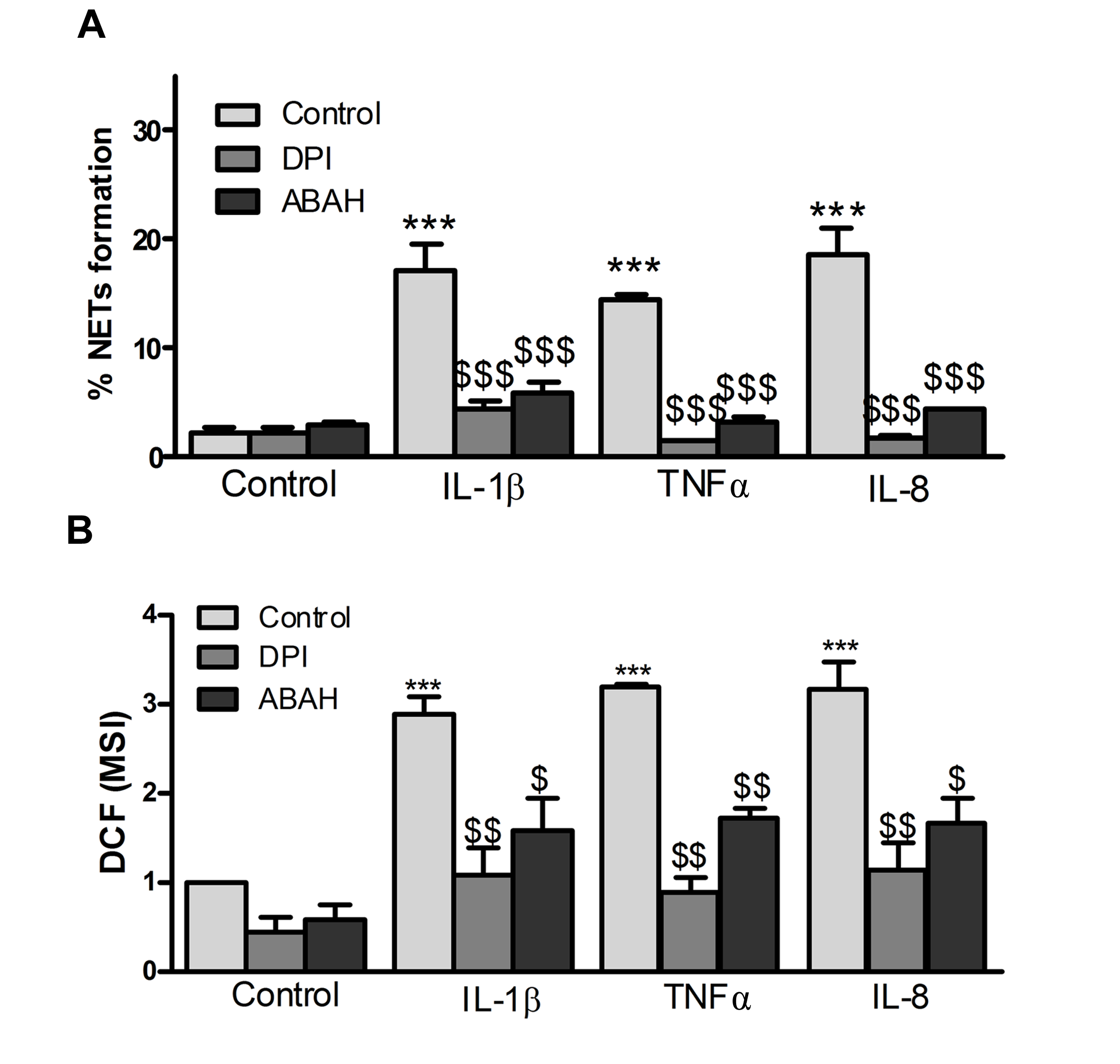

Supplement: Figure S2 — IL-8, TNFα or IL-1β induced NETs release and free radical generation. (A) Bar diagram representing NETs release following stimulation of PMNs from healthy subjects with recombinant IL-8, TNFα or IL-1β (***p<0.001 vs control; $$$p<0.001 vs stimulator). (B) Bar diagram representing free radical generation following stimulation with recombinant IL-8, TNFα or IL-1β (**p<0.01, ***p<0.001 vs control; $p<0.05, $$p<0.01 vs stimulator). (TIF) [file pone.0048111.s002.tif]

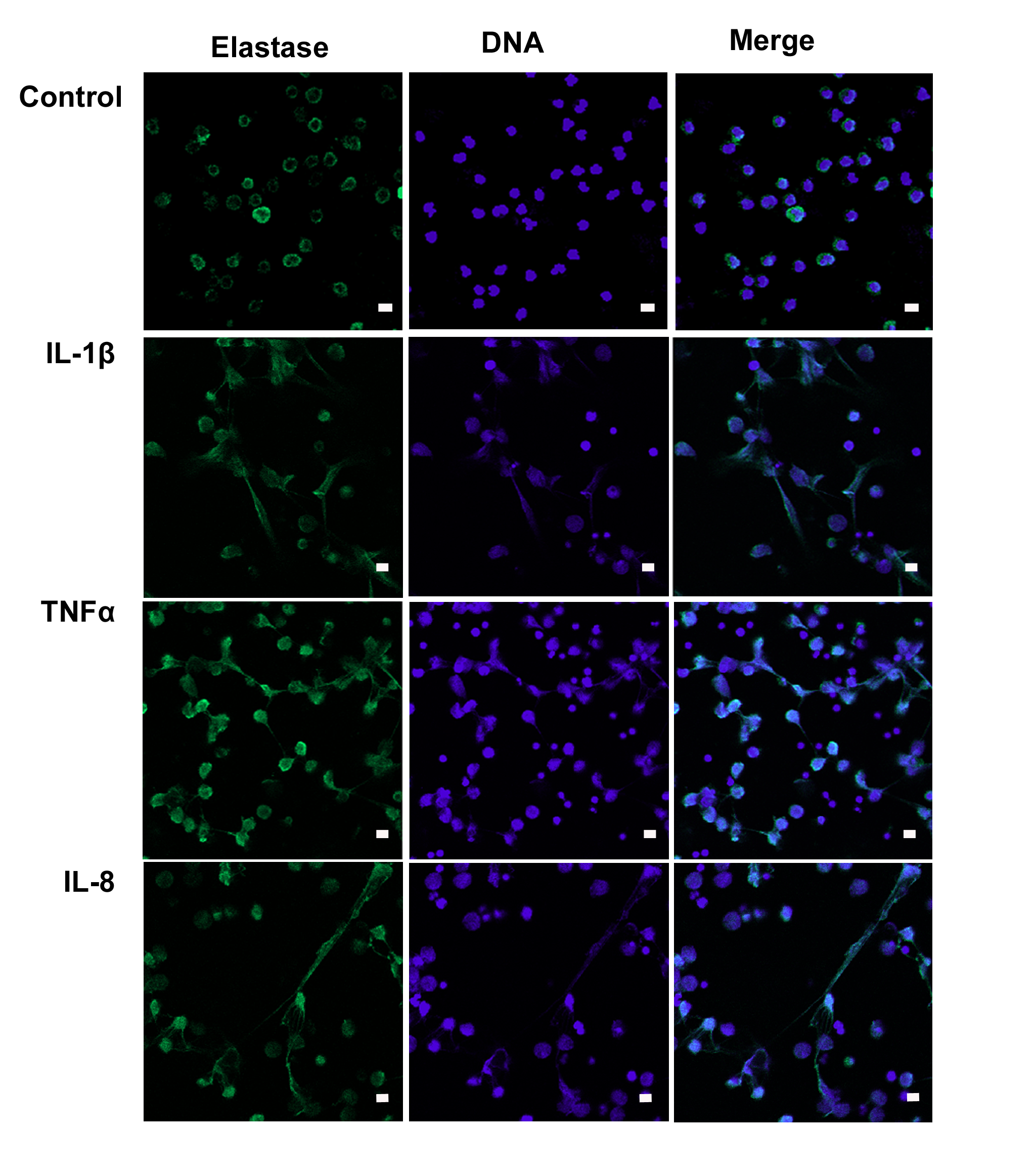

Supplement: Figure S3 — Immuno-histochemical characterization of IL-8, TNF or IL-1β induced NETs release in neutrophils. Resting neutrophils stained with elastase antibody conjugated with AF 488 (green) and Hoechst 33258 (blue) showing multilobed nuclei and punctate elastase. Neutrophils treated with IL-8, TNFα or IL-1β led to the formation of NETs (Bar 10 µm). (TIF) [file pone.0048111.s003.tif]
